# Supplementary material for: Venomix: a simple bioinformatic pipeline for identifying and characterizing toxin gene candidates from transcriptomic data
Source: PeerJ. 2018 Jul 31;6:e5361. doi: 10.7717/peerj.5361 (PMC6074769; doi:10.7717/peerj.5361)
Supplement: Supplemental Information 6 [file peerj-06-5361-s006.gz › FinalOutput_1E-6/U24-ctenitoxin-Pn1a_1/finaltree.pdf]

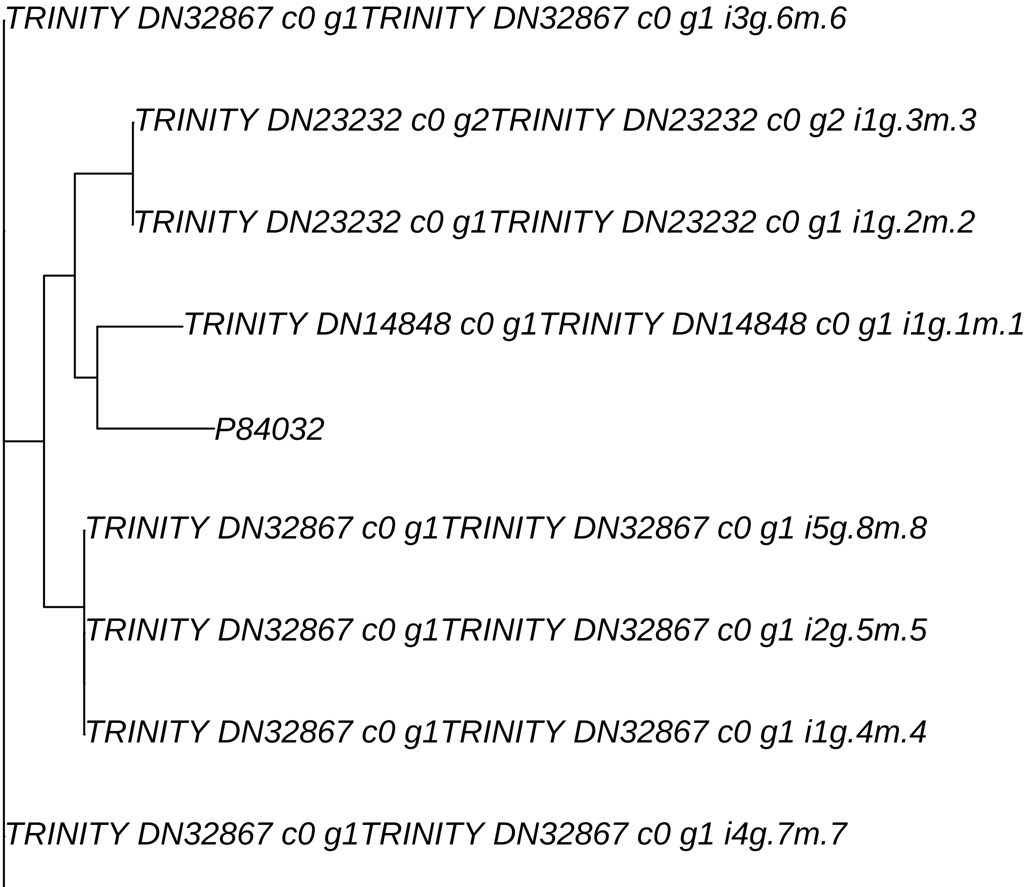

TRINITY DN32867 c0 g1TRINITY DN32867 c0 g1 i3g.6m.6
TRINITY DN23232 c0 g2TRINITY DN23232 c0 g2 i1g.3m.3
TRINITY DN23232 c0 g1TRINITY DN23232 c0 g1 i1g.2m.2
TRINITY DN14848 c0 g1TRINITY DN14848 c0 g1 i1g.1m.1
P84032
TRINITY DN32867 c0 g1TRINITY DN32867 c0 g1 i5g.8m.8
TRINITY DN32867 c0 g1TRINITY DN32867 c0 g1 i2g.5m.5
TRINITY DN32867 c0 g1TRINITY DN32867 c0 g1 i1g.4m.4
TRINITY DN32867 c0 g1TRINITY DN32867 c0 g1 i4g.7m.7
TRINITY DN32867 c0 g1TRINITY DN32867 c0 g1 i6g.9m.9
